# Does Chronic Cannabis Use Impact Risky Decision-Making: An Examination of fMRI Activation and Effective Connectivity?

**DOI:** 10.3389/fpsyt.2020.599256

**Published:** 2020-11-27

**Authors:** David R. Raymond, Adrian Paneto, Karmen K. Yoder, Brian F. O'Donnell, Joshua W. Brown, William P. Hetrick, Sharlene D. Newman

**Affiliations:** ^1^Department of Psychological and Brain Sciences, Indiana University Bloomington, Bloomington, IN, United States; ^2^Department of Counseling Psychology, Indiana University Bloomington, Bloomington, IN, United States; ^3^Department of Radiology, Indiana University Medical School, Indianapolis, IN, United States; ^4^Program in Neuroscience, Indiana University Bloomington, Bloomington, IN, United States; ^5^Department of Psychology, Alabama Life Research Institute, University of Alabama Tuscaloosa, Tuscaloosa, AL, United States

**Keywords:** cannabis, decision-making, effective connectivity, fMRI, reward, risk

## Abstract

With the increase in use of cannabis and its shifting legal status in the United States, cannabis use has become an important research focus. While studies of other drug populations have shown marked increases in risky decision-making, the literature on cannabis users is not as clear. The current study examined the performance of 17 cannabis users and 14 non-users on the Balloon Analog Risk Task (BART) using behavioral, fMRI and effective connectivity methods. Significant attenuation was found in a functional pathway projecting from the dorsal anterior cingulate cortex (dACC) to the nucleus accumbens (NAc) in cannabis users compared to non-using controls as well as decreases in risk-taking behaviors. These findings suggest that cannabis users may process and evaluate risks and rewards differently than non-users.

## Introduction

Cannabis (CB) use has been on the rise in recent years, in part due to the drug's increased acceptance and shifting status from an illegal to a legal drug in some US states. Cannabis is the most used illicit drug in the United States and thus is an important area of study. Delta-9-tetrahydrocannabinol (THC) is the psychoactive component of CB and has been linked to depression (1) and psychotic disorders, including schizophrenia (2–4). Heavy CB use has also been linked to poorer neurocognitive functioning (5–8).

While the chronic use of alcohol and other drugs of addiction have been associated with increased risk-taking behaviors and poor inhibitory control (9), CB use has not consistently been found to be linked to increased risk-taking (10, 11). For example, Gilman et al. (12) found that increased risk-taking behavior in CB users depended on stimulus type with greater risk-taking observed when the rewards were social, health/safety, and ethical factors but not when the rewards were monetary. A study by Vivas et al. (11) found that CB use actually enhanced inhibitory control compared to non-users. Another study used transcranial direct current stimulation (tDCS) to stimulate the left and right dorsal lateral prefrontal cortex (DLPFC) in chronic cannabis users and controls and found that chronic cannabis users made more conservative decisions than controls during sham stimulation (placebo) but during active stimulation of the right DLPFC, controls made more conservative decisions while activations of both right and left DLPFC in cannabis users led to increased risk-taking (13). Additionally, Wesley et al. (14) found that cannabis users performed worse on a version of the Iowa Gambling Task than controls and that during that cannabis users showed significantly less activation in response to loss during the strategy planning phase of the task, namely in the anterior cingulate cortex (ACC), medial frontal cortex, precuneus, superior parietal lobe, occipital lobe and cerebellum. These results suggest various disturbances in regions of executive function, as well as in certain properties like reward salience, in chronic cannabis users which do not paint a clear picture of what these differences could mean.

Task-based activation and resting state functional MRI studies have shown altered activity and connectivity between key regions associated with risky decision-making in CB users; however, there are inconsistencies regarding how the connectivity varies across studies. The primary regions involved in risky decision making include those related to affective processing of stimuli (anterior insula and ventral striatum, including the nucleus accumbens) and integrating cognitive and affective information (medial prefrontal cortex, including the anterior cingulate) (15–17). Cousijn et al. (16) found that the amount of weekly CB use was positively related to activation in the right anterior insula, right ventral striatum and ventrolateral prefrontal cortex during an Iowa Gambling Task. Additionally, Lichenstein et al. (18) reported attenuated functional connectivity (FC) between the nucleus accumbens (NAc) and the medial prefrontal cortex (mPFC) in CB users. Fischer et al. (19) found a similar result, such that there was reduced resting state functional connectivity (rsFC) between the NAc and PFC in patients with CB use disorder and schizophrenia. However, Filbey et al. (20) found increased FC between the NAc and the ACC when CB users were viewing CB use cues. While previous studies do show activation and connectivity differences between CB users and non-users there are still a limited number of studies and there is still some inconsistency with regard to the directionality of connectivity differences.

The current study uses the Balloon Analog Risk Task (BART) to examine risk-taking behavior in CB users. The BART is a gambling task designed as a behavioral measure of risk taking which requires participants to inflate a balloon more and more for money while risking the balloon exploding and losing their money. The BART has been used to investigate the relationship between risk-taking and decision-making in various drug use groups. Researchers have found that number of balloon inflations (more inflations is equivalent to higher levels of risk-taking) is increased in nicotine smokers relative to non-smokers (21) and is positively correlated with severity of polysubstance use (22). Alternatively, number of balloon inflations was found to be negatively correlated with long-term alcohol use in a 2013 study by Campbell et al. (23) While the BART has been used to study risk-taking and reward processing in different substance users, few studies have investigated differences in BART performance in CB users and those that have found no differences in BART performances between CB users and non-users, but did find negative correlations between Cannabis Use Disorder symptoms and CB use severity with performance in other risk-taking paradigms, namely the IGT (24, 25). The current study uses a modified version of the BART which utilizes parametric modulation to generate more precise representations of risk-taking in hopes of clarifying the disparity in results.

The current study also uses effective connectivity analysis to examine the connectivity differences within the reward network between CB users and non-users. Effective connectivity tests an *a priori* defined model containing directed connections (26). Here a model that included a connection from the dorsal ACC (dACC) to the NAc, the anterior insula to the NAc, and the anterior insula to the dACC was tested. This model was based on previous studies that show a directed glutamatergic connection between the dACC and NAc which plays an important role in modulating the addicted brain's response to rewards (27). A number of studies have proposed a connection between the ACC and insula (28, 29), with White and colleagues finding that the activation of the insula precedes that of the ACC suggesting a potential directed connection from the insula to the ACC. Finally, previous work using effective connectivity during cue-elicited incentive anticipation, a component of reward processing which is shown to be maladaptive in people who are addicted to drugs (30), has also suggested a directed connection from the insula to the NAc (15). It was predicted that this reward network would be disrupted in CB users while performing a risky decision task.

## Methods

### Participants

A total of 40 participants took part in the study. Subjects were recruited by local advertisements. After detailed description of the study, written and verbal informed consent was obtained from each participant. All subjects were required to be 18 years or older. Subjects were asked to refrain from alcohol or CB use the day prior to the MRI scan. The research protocol was approved by Indiana University's Institutional Review Board for the protection of human subjects.

### Inclusion/Exclusion Criteria

All participants had to be free of psychological disorders (with the exception of cannabis use disorder for the CB group), free of any neurological disorder, head trauma with loss of consciousness > 10 min, learning disability, contraindication to MRI, be between the ages of 18 and 30 years, not a user of illicit drugs (other than CB), and have abstained from CB and alcohol use for at least 12 h prior to the scan. Participants completed a battery of assessments including the Structured Clinical Interview for DSM-IV-TR (SCID-IV-TR), Research Version (31); a written drug use questionnaire; the short Michigan alcohol screening test (SMAST); the Cannabis Use Disorder Identification Test (CUDIT). The control subjects had no history of substance dependence, and no use of CB in the past 3 months. The CB group were not required to have a diagnosis of CUD. The group characteristics include: [1] an average age of CB initiation of 16.5 ± 1.9 years; [2] used an average of 5.2 ± 2.1 days/week; [3] used an average of 11.4 ± 7.4 joints/bowls/week; and 4) of the 13 who have used wax, 11 had used in the past 6 months. Eight were lost to existing mood disorders (depression and/or anxiety) and an additional one was lost to excessive subject motion during scanning. This left 14 control non-users (6 males, age 23.5 ± 4) and 17 CB users (8 males, age 21.2 ± 3). Groups did not significantly differ in age, sex, days since last alcohol use at the time of screening or SMAST score (*p* > 0.1) (see Table 1).

**Table 1:** Demographics.

	**Controls**	**CB Users**	**2-tailed *t*-test *p*-value**
*n*	14	17	
#Males	6	8	
Age	23.5 ± 4	21.2 ± 3	0.093
Average days since last CB use (prior to scan)		1.3 ± 1 days	
Average days since last alcohol use (prior to scan)	6.4 ± 5.5	3.6 ± 2.8	0.42
CUDIT	0.15 ± 0.4	13.4 ± 4.8	<0.0001
SMAST	0.5 ± 1	0.29 ± 0.7	0.58

### Procedure

Potential participants were contacted via telephone and underwent a preliminary screening process. If the potential participant qualified for the study, they were scheduled for a testing day. On this day, participants arrived at the laboratory space and after signing a consent form, completed a variety of surveys and batteries about demographics and drug and alcohol use. After completing these surveys, researchers examined the results to ensure the participants still qualified for the MRI scans. If the participants qualified, a 2nd day was scheduled in which participants underwent the MRI tests while completing the BART. After the MRI scan, participation was complete and participants were compensated for their time and their performance at the BART.

### BART Task

The BART design used in this study was modeled from previous imaging design (32) and was administered in two, 8-min blocks during fMRI data collection. As participants continued to inflate in pursuit of greater reward, the probability of an explosion increased parametrically. Participants were informed that higher winnings during the task would yield in bonus monetary reward for participation in the study in order to incentivize participation and mimic real-world risk-reward decision making.

**Figure 1:**
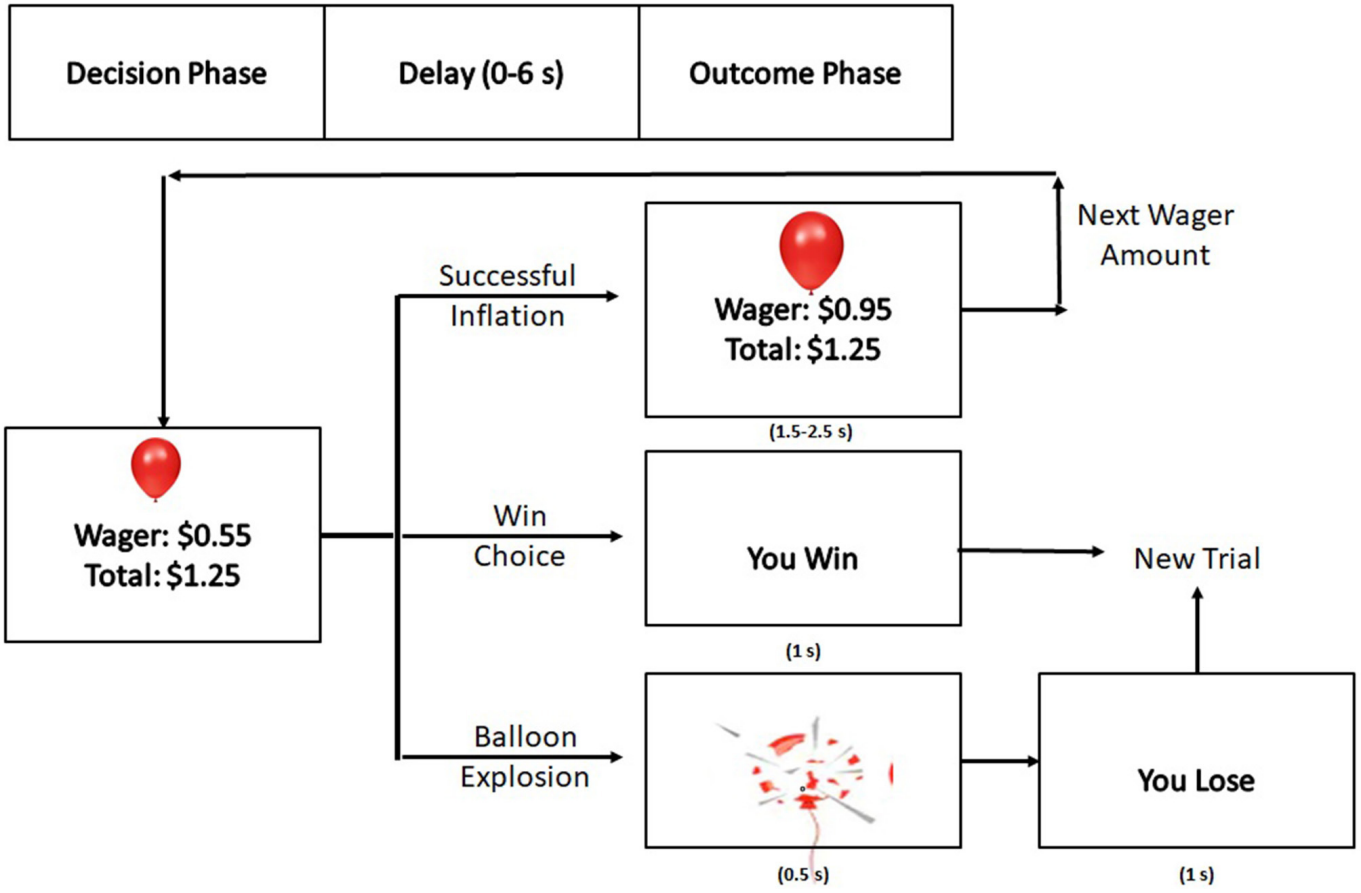
A Depiction of the typical trial in the BART task.

Each block began and ended with a 30 s, white fixation cross (“+”) on a black background in order to establish a baseline for activity. At the beginning of each trial, the screen displayed the image of a purple balloon above a small, green rectangle which indicated that the participant should make a decision. This rectangle was above the participant's current wager amount for that balloon and the participant's total winnings earned for that block at that point in time (Figure 1). At this point, the participant had unlimited time to choose inflation or to “win” and add the current wager to their total winnings. After this decision was made, there was a delay between 0 and 6 s before the outcome (balloon explosion, successful inflation, or “You Win!”) was displayed. The winning display was present for 1 s. If the inflation was successful, then the decision rectangle would be red for either 1.5, 2, or 2.5 s, indicating that a decision could not be made. Once the rectangle became green again, the participant could make a decision. If the inflation was unsuccessful, an exploded balloon was presented for 0.5 s followed by the text “You Lose!,” which was present for 1 s. After either a win or a loss, the screen was blank for 2, 3, or 4 s until a new trial display was presented.

Along with the baseline monetary compensation for time and participation in this study, participants were rewarded with additional funds based on their performance on the BART (and additional 50% of total earnings over two trials of the BART).

### MRI Acquisition and Analysis

Image acquisition was performed on a 3T Siemens Prisma MRI scanner and using a 64-channel head coil. Foam pads were used to minimize head motion for all participants. High-resolution T1-weighted anatomical images were acquired in the sagittal plane using an MP-RAGE sequence [TR = 2.4 s; TE = 2.36 ms; inversion time = 1.0 s; flip angle 8°; imaging matrix = 320 × 320; 256 slices; voxel size = 0.7 × 0.7 × 0.7 mm^3^]. Functional BOLD data for each participant was collected in two blocks using a gradient echo T2-weighted echo planar imaging sequence [TR = 2.0 s; TE = 0.25 s; flip angle 70°; imaging matrix = 64x220; 35 slices; voxel size = 3.4 × 3.4 × 3.8 mm^3^; 0-mm gap; 240 volumes].

MRI data were processed and analyzed using SPM5 [University College London; (33)]. The preprocessing steps that were applied to the functional MRI data included: slice timing correction, motion correction using a rigid body realignment algorithm, co-registration, spatial normalization using the MNI template and each person's T1 scan, and smoothing with the Gaussian kernel filter of 8 mm. The final voxel size after normalization was 2 × 2 × 2 mm^3^. The amount of head motion was closely examined and no subject showed excessive movements > 1 mm.

Event-related responses were analyzed using a general linear model (GLM) with 9 experimental condition regressors, 2 constants, and 6 motion regressors. Five of experimental condition conditions included: the choice to inflate the balloon (ChooseInflate); the choice to stop inflating the balloon (ChooseWin); the losing/balloon explosion outcome (ExplodeOutcome); the successful inflation outcome (Successful Inflate); and the winning outcome (WinOutcome). The remaining conditions were four parametric modulators to identify brain regions where activation was positively or negatively correlated with the probability of explosion: ChooseInflate^*^P(explode), ChooseWin^*^P(explode), WinOutcome^*^P(explode), and ExplodeOutcome^*^P(explode). The ChooseInflate^*^P(explode) is referred to here is the risk-taking condition that was examined in this analysis. Activation threshold was set at *p* < 0.001 with an extent of 150 voxels to correct for multiple corrections.

### Effective Connectivity Modeling and Analysis

Effective connectivity analyses, or the average change in BOLD activity in one ROI as influenced by a different ROI, was conducted using Structural Equation Modeling (SEM) and performed using SPSS (25, IBM Corporation) and AMOS (25, IBM Corporation). ROIs and the directionality of their connections were determined a priori and as described in the introduction. The ROIs and the network constructed were determined by the wealth of evidence which associate and incorporate the dACC, NAc, and insula with reward and decision-making processes (15, 34). The beta weights from the GLM fMRI analysis for the risk-taking condition were extracted for each ROI. ROIs were determined using the group analysis (collapsed across CB user and non-user groups). Those beta weights were used as input into the predesigned model. A multi-group path analysis was performed in AMOS using critical ratios for differences between parameters to test pair-wise coefficient differences. Coefficients for each path within the network were generated using multi-group path analysis.

## Results

### Behavioral Results

When examining the performance differences between groups, the control group won more money than did the CB user group, see Table 2. No other measures were found to be significantly different.

**Table 2:** Behavioral results.

	**CB users**	**Non-users**	**2-tailed *t*-test *p*-value**
Winnings	20.7 ± 5.6	25.9 ± 7.3	0.03
Trials completed	34.5 ± 4.5	33.9 ± 5.9	0.8
Inflations	167.5 ± 23	171.9 ± 14.2	0.5
Wins	21.1 ± 5.7	21.6 ± 7.5	0.8
Explosions	13.4 ± 5.2	12.3 ± 3.3	0.5

### fMRI Results

**Figure 2:**
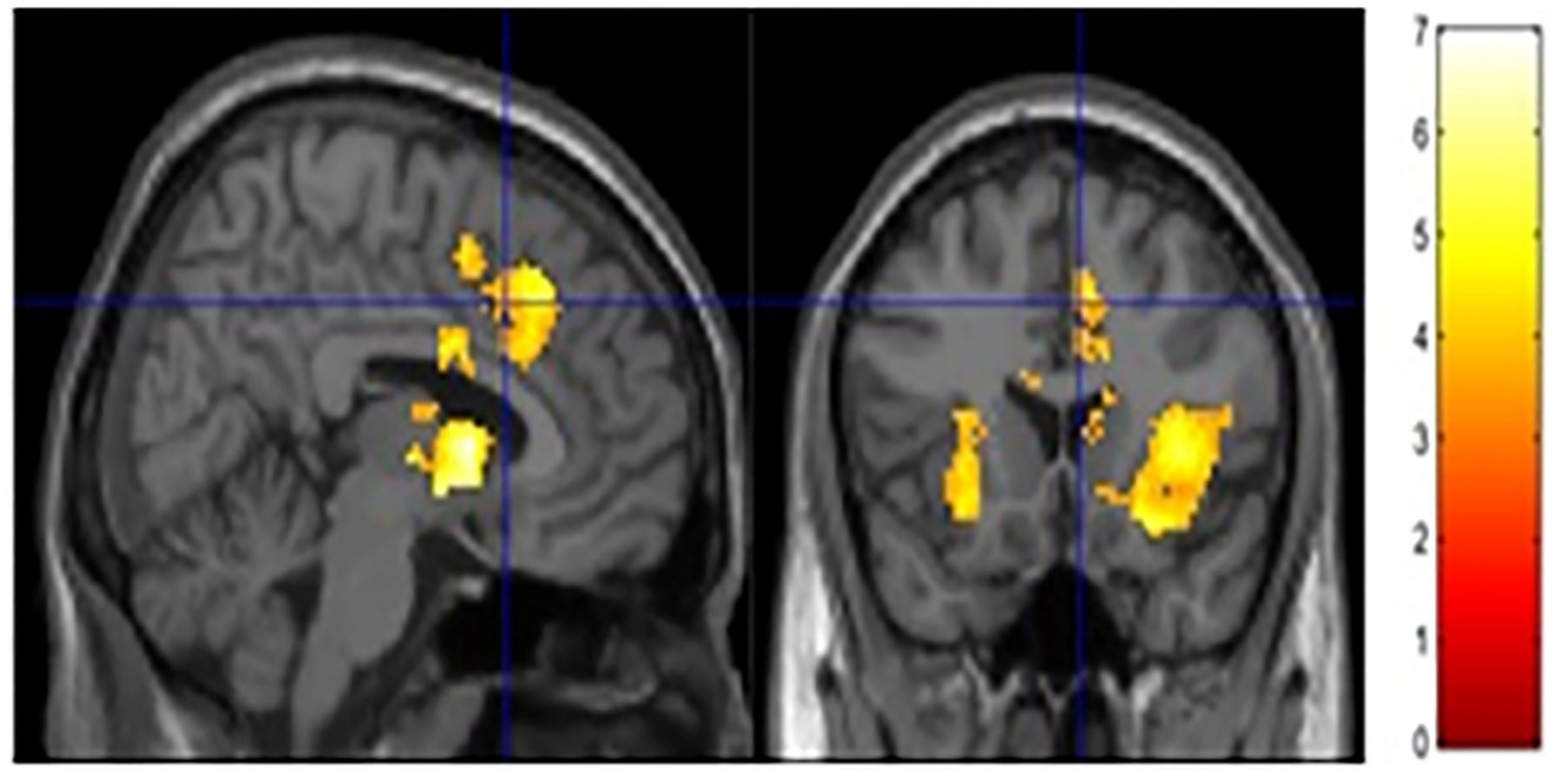
Risk activity collapsed across groups. Significant activity was observed in the ACC, NAc and bilateral insula.

The current study focused on activation related to risk-taking. Risk-taking was examined by parametrically modulating the decision to inflate with the probability of balloon explosion. An analysis of activation related to risk taking behaviors collapsed across both groups showed significant activation in regions typically associated with risky decision making and reward seeking, such as the dACC, NAc, and insula (see Figure 2 and Table 3). However, no significant group differences were observed after correcting for multiple comparisons (see Supplementary Material for other analyses).

**Table 3:** fMRI activation.

**Region**	**BA**	***k***	***z***	***x, y, z***
R. Ventral striatum		2,191	5.33	6, 8, 4
R. insula	13		5.09	36, 22, 4
Anterior Cingulate	32	456	4.75	8, 28, 42
R. Ventral striatum		307	4.53	14, −14, 20
R. Insula	13	256	4.38	34, −36, 24
L. Insula	13	434	4.27	−28, 22, 0
L. Insula	13	177	4.16	−28, −34, 38
R. Precentral	4	182	4.13	26, −16, 42

### Effective Connectivity

**Figure 3:**
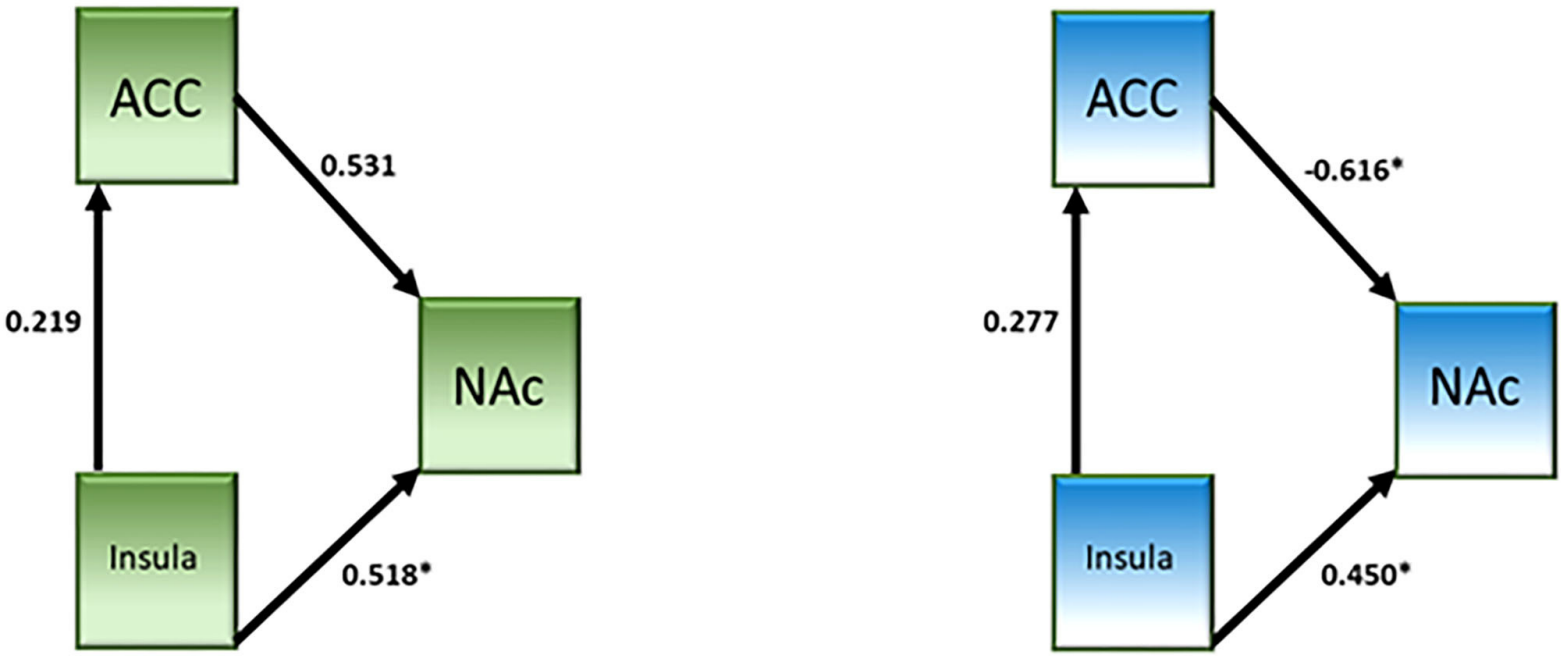
Effective connectivity network analysis results from the cannabis use group (left, green) and the control group (right, blue).

A network analysis was performed. The unconstrained model had a good fit (χ^2^ = 0.64, *p* = 0.42, CFI = 1.000, IFI = 1.019). Both groups showed a significant connection from the insula to NAc (see Table 4 and Figure 3). Neither group showed a significant connection from the insula to the dACC. The connection from the dACC to the NAc was found to be significant for the non-user group but not the CB user group. When directly comparing the parameter estimates, the connection from the dACC to the NAc was found to be significantly different between groups [z-score = −3.121, *p* < 0.05]. Additionally, the connectivity from the dACC to the NAc was negative in the non-user group, suggesting that the dACC has an inhibitory effect on the NAc in non-CB users but not CB users.

**Table 4:** Effective connectivity parameter estimates.

			**Estimate**	**S.E**.	**C.R**.	***p***
**CB users**						
dACC	< ---	Insula	0.219	0.176	1.248	0.212
NAC	< ---	Insula	0.518	0.228	2.271	0.023
NAC	< ---	dACC	0.531	0.311	1.708	0.088
**Non-users**						
dACC	< ---	Insula	0.277	0.234	1.186	0.236
NAC	< ---	Insula	0.45	0.175	2.579	0.01
NAC	< ---	dACC	−0.616	0.196	−3.14	0.002

## Discussion

The primary goal of the current study was to explore the hypothesis that CB interacts with the brain network responsible for risky decision making. The results show that in the group of high functioning chronic CB users the effect is minimal in that there were no fMRI-measured brain activation differences compared to non-using controls when performing the BART task. While there were no brain activation differences between groups, effective connectivity analysis revealed significantly attenuated connectivity between the dACC to the NAc in CB users compared to controls. The results may also suggest that CB users may be more risk *averse* than non-using controls.

Previous studies have found that drug users tend to be more impulsive and risk-taking (9). However, the results reported in the current study suggest that CB users may actually be more risk averse than the non-user group. The users won significantly less money even though they had a similar number of explosions and win trials. The reason for the decreased winnings is likely due to prematurely stopping inflations. These findings are contrary to previous studies of drug users. A potential explanation is that CB users may be engaging in more deliberative, as opposed to impulsive, risk-taking behavior. Whiteside and Lynam (35) identified distinct factors of impulsive-like behavior - urgency, (lack of) premeditation, (lack of) perseverance, and sensation seeking. It has also been argued risky decision-making can be conducted using deliberative procedures as well. These different factors of impulsive decision-making elicit different patterns of brain activation [e.g., (36)] with the medial prefrontal cortex, including the dACC, being involved to monitor or inhibit impulsive decisions. Given that the connectivity between the dACC and NAc is lesser in CB users compared to non-users, this may suggest that this pathway either does not operate efficiently or that a more deliberative strategy is preferred by these high functioning CB users. While speculative, some support for this hypothesis can be found by an increased involvement of the right lateral prefrontal cortex for CB users compared to non-users at a lower threshold (see Supplementary Material) which has been linked to more deliberative processing (37). Other support can be found in the research on drug use and driving (38, 39). For example, MacDonald et al. (38) found that cannabis users were more cautious when driving under the influence or refrained from driving altogether which was the opposite finding for cocaine users. Another potential explanation for the behavioral finding of potential risk aversion may be related to the use of monetary reward. Gilman et al. (12) failed to observe differences between CB users and non-users during a financial risk-taking task but did observe differences when using social stimuli. It may be that the decreased salience of the monetary reward used in the current study is disincentivizing the CB users to take more risks (i.e., inflate more), or put another way, the monetary reward does not lead to the use of the impulsive strategy but the deliberative one. These effects could be caused by the chronic use of cannabis which makes cannabis a more salient reward than money.

The connectivity from the dACC to the NAc was attenuated in CB users; this attenuation was linked to risky decision-making processes as it was observed for the risk parametric modulator. In addition the connectivity was inhibitory in non-users but not in CB users. A study by Lichentstein et al. (18) showed increased functional connectivity between the NAc and dACC in CB users in response to cannabis-related cues relative to neutral cues. The differences between the Lichentstein et al. study and the current study may account for the differences in results, namely the task and the stimuli (money vs. cannabis). However, both studies show that the connectivity between the dACC and NAc are impacted by chronic CB use. The core of the NAc has been shown to be anatomically connected to the dACC (40). Phasic dopamine release in the core, but not the shell, has been observed following reward-predictive cues (41) and that dopamine release is related to the subjective reward value of the cue (42–44). This suggests that the subjective value of the reward plays a role in how individuals make decisions regarding said rewards, which may account for group differences observed in the current study as well as previous studies showing differences in reward processing as a function of the reward in CB users. Additionally, it may be salience of loss that drives the behavior of CB users, like was seen in Wesley et al. (14). It will be important in future studies to assess the subjective reward value in participants in order to more fully understand risky decision-making and reward processing.

### Limitations

There are some limitations of the current study. First, number of participants is small (*N* = 31) and therefore limits the conclusions that can be drawn. The limited sample size may explain the lack of significance in the between group analysis of fMRI activation. Another potential limitation is that we were unable to examine sex differences due to the small sample size. Previous studies have shown sex differences in neurochemistry when examining chronic CB users; therefore, it is important to further explore those differences when examining risky decision-making. Additionally, some have complained that the BART as well as similar tasks do not effectively engage risk-like behavior due to its repetitive nature; participants “figuring out” the task and how to maximize winnings and then the task no longer assesses risk. Finally, in an attempt to ensure that participants were not intoxicated during the scan they were asked to abstain prior to the session. A recent study suggests that cognitive deficits observed in the abstention period used (<72 h) could be due to either withdrawal or residual effects of acute use (45).

### Conclusions

This preliminary study examining risky decision-making suggests, while minimal, that CB use is associated with functional connectivity from the dACC to the NAc. The decrease in connectivity and the switch between inhibitory to excitatory connectivity may suggest the use of different strategies, or differences in the subjective value of the reward between groups. Further research is necessary to disentangle these possibilities and to replicate the current findings.

## Data Availability Statement

The data discussed in this article are available upon request. Please send inquiries to the corresponding author.

## Ethics Statement

The studies involving human participants were reviewed and approved by Institutional Review Board. The patients/participants provided their written informed consent to participate in this study.

## Author Contributions

DR collected and analyzed data and prepared the manuscript. AP designed the study and collected and analyzed data. KY designed the study and prepared the manuscript. BO'D designed the study and recruited subjects for data collection. JB designed the study and prepared the manuscript. WH designed the study. SN designed and managed the study, analyzed the data, and prepared the manuscript. All authors contributed to the article and approved the submitted version.

## Conflict of Interest

The authors declare that the research was conducted in the absence of any commercial or financial relationships that could be construed as a potential conflict of interest.

## References

[1] Wright NE Scerpella D Lisdahl KM. Marijuana use is associated with behavioral approach and depressive symptoms in adolescents and emerging adults. PLoS ONE. (2016) 11:e0166005. 10.1371/journal.pone.0166005 27835662 PMC5106002

[2] Andréasson S Engström A Allebeck P Rydberg U Cannabis and schizophrenia A longitudinal study of Swedish conscripts. Lancet. (1987) 330:1483–6. 10.1016/S0140-6736(87)92620-1 2892048

[3] Arseneault L Cannon M Poulton R Murray R Caspi A Moffitt TE. Cannabis use in adolescence and risk for adult psychosis: longitudinal prospective study. BMJ. (2002) 325:1212–3. 10.1136/bmj.325.7374.1212 12446537 PMC135493

[4] Van Os J Bak M Hanssen M Bijl RV De Graaf R Verdoux H. Cannabis use and psychosis: a longitudinal population-based study. Am J Epidemiol. (2002) 156:319–27. 10.1093/aje/kwf043 12181101

[5] Crean RD Crane NA Mason BJ. An evidence based review of acute and long-term effects of cannabis use on executive cognitive functions. J Addict Med. (2011) 5:1. 10.1097/ADM.0b013e31820c23fa 21321675 PMC3037578

[6] Doss MK Weafer J Gallo DA de Wit H. Δ9-tetrahydrocannabinol at retrieval drives false recollection of neutral and emotional memories. Biol Psychiatr. (2018) 84:743–50. 10.1016/j.biopsych.2018.04.020 29884456

[7] Meier MH Caspi A Ambler A Harrington H Houts R Keefe RS. Persistent cannabis users show neuropsychological decline from childhood to midlife. Proc Natl Acad Sci USA. (2012) 109:E2657–64. 10.1073/pnas.1206820109 22927402 PMC3479587

[8] Ranganathan M Radhakrishnan R Addy PH Schnakenberg-Martin AM Williams AH Carbuto M. Tetrahydrocannabinol (THC) impairs encoding but not retrieval of verbal information. Prog Neuro-Psychopharmacol Biol Psychiatr. (2017) 79:176–83. 10.1016/j.pnpbp.2017.06.019 28642081

[9] Stephan RA Alhassoon OM Allen KE Wollman SC Hall M Thomas WJ. Meta-analyses of clinical neuropsychological tests of executive dysfunction and impulsivity in alcohol use disorder. Am J Drug Alcohol Abuse. (2017) 43:24–43. 10.1080/00952990.2016.1206113 27712350 PMC6462408

[10] Broyd SJ van Hell HH Beale C Yuecel M Solowij N. Acute and chronic effects of cannabinoids on human cognition—a systematic review. Biol Psychiatr. (2016) 79:557–67. 10.1016/j.biopsych.2015.12.002 26858214

[11] Vivas AB Estevez AF Moreno M Panagis G Flores P. Use of cannabis enhances attentional inhibition. Clin Experi. (2012) 27:464–9. 10.1002/hup.2248 22859379

[12] Gilman JM Calderon V Curran MT Evins AE. Young adult cannabis users report greater propensity for risk-taking only in non-monetary domains. Drug Alcohol Depen. (2015) 147:26–31. 10.1016/j.drugalcdep.2014.12.020 25577478 PMC4297698

[13] Boggio PS Zaghi S Villani AB Fecteau S Pascual-Leone A Fregni F. Modulation of risk-taking in marijuana users by transcranial direct current stimulation (tDCS) of the dorsolateral prefrontal cortex (DLPFC). Drug Alcohol Depen. (2010) 112:220–5. 10.1016/j.drugalcdep.2010.06.019 20729009

[14] Wesley MJ Hanlon CA Porrino LJ. Poor decision-making by chronic marijuana users is associated with decreased functional responsiveness to negative consequences. Psychiatr Res. (2011) 191:51–9. 10.1016/j.pscychresns.2010.10.002 21145211 PMC3125637

[15] Cho YT Fromm S Guyer AE Detloff A Pine DS Fudge JL. Nucleus accumbens, thalamus and insula connectivity during incentive anticipation in typical adults and adolescents. Neuroimage. (2013) 66:508–21. 10.1016/j.neuroimage.2012.10.013 23069809 PMC3949208

[16] Cousijn J Wiers RW Ridderinkhof KR van den Brink W Veltman DJ Porrino LJ. Individual differences in decision making and reward processing predict changes in cannabis use: a prospective functional magnetic resonance imaging study. Addict Biol. (2013) 18:1013–23. 10.1111/j.1369-1600.2012.00498.x 22994937

[17] Li X Lu ZL D'Argembeau A Ng M Bechara A. The Iowa gambling task in fMRI images. Human Brain Mapp. (2010) 31:410–23. 10.1002/hbm.20875 19777556 PMC2826566

[18] Lichenstein SD Musselman S Shaw DS Sitnick S Forbes EE. Nucleus accumbens functional connectivity at age 20 is associated with trajectory of adolescent cannabis use and predicts psychosocial functioning in young adulthood. Addiction. (2017) 112:1961–70. 10.1111/add.13882 28547854 PMC5633503

[19] Fischer AS Whitfield-Gabrieli S Roth RM Brunette MF Green AI. Impaired functional connectivity of brain reward circuitry in patients with schizophrenia and cannabis use disorder: effects of cannabis and THC. Schizophrenia Res. (2014) 158:176–82. 10.1016/j.schres.2014.04.033 25037524 PMC4778557

[20] Filbey FM Dunlop J. Differential reward network functional connectivity in cannabis dependent and non-dependent users. Drug Alcohol Depend. (2014) 140:101–11. 10.1016/j.drugalcdep.2014.04.002 24838032 PMC4349558

[21] Lejuez CW Aklin WM Zvolensky MJ Pedulla CM Evaluation of the Balloon Analogue Risk Task (BART) as a predictor of adolescent real-world risk-taking behaviours. J Adolescen. (2003) 26:475–9. 10.1016/S0140-1971(03)00036-8 12887935

[22] Hopko DR Lejuez CW Daughters SB Aklin WM Osborne A Simmons BL Construct validity of the balloon analogue risk task (BART): relationship with MDMA use by inner-city drug users in residential treatment. J Psychopathol Behav Assessment. (2006) 28:95–101. 10.1007/s10862-006-7487-5

[23] Campbell JA Samartgis JR Crowe SF. Impaired decision making on the balloon analogue risk task as a result of long-term alcohol use. J Clin Experi Neuropsychol. (2013) 10:1071–81. 10.1080/13803395.2013.856382 24215387

[24] Gonzalez R Schuster RM Mermelstein RJ Vassileva J Martin EM Diviak KR Performance of young adult cannabis users on neurocognitive measures of impulsive behavior and their relationship to symptoms of cannabis use disorders. J Clin Experi Neuropsychol. (2012) 34:962–76. 10.1080/13803395.2012.703642 PMC3488122 22882144

[25] Crane NA Schuster RM Gonzalez R. Preliminary evidence for a sex-specific relationship between amount of cannabis use and neurocognitive performance in young adult cannabis users. J Int Neuropsychol Soc. (2013) 19:1009. 10.1017/S135561771300088X 23962414 PMC3895398

[26] Lindquist MA The statistical analysis of fMRI data. Statist Sci. (2008) 23:439–64. 10.1214/09-STS282

[27] Kalivas PW Volkow ND. New medications for drug addiction hiding in glutamatergic neuroplasticity. Mol Psychiatr. (2011) 16:974. 10.1038/mp.2011.46 21519339 PMC3192324

[28] Klumpp H Angstadt M Phan KL. Insula reactivity and connectivity to anterior cingulate cortex when processing threat in generalized social anxiety disorder. Biol Psychol. (2012) 89:273–6. 10.1016/j.biopsycho.2011.10.010 22027088 PMC3260042

[29] White TP Joseph V Francis ST Liddle PF. Aberrant salience network (bilateral insula and anterior cingulate cortex) connectivity during information processing in schizophrenia. Schizophrenia Res. (2010) 123:105–15. 10.1016/j.schres.2010.07.020 20724114

[30] Zilverstand A Huang AS Alia-Klein N Goldstein RZ. Neuroimaging impaired response inhibition and salience attribution in human drug addiction: a systematic review. Neuron. (2018) 98:886–903. 10.1016/j.neuron.2018.03.048 29879391 PMC5995133

[31] First MB Spitzer RL Gibbon M Williams JB Structured Clinical Interview for DSM-IV-TR Axis I Disorders, Research Version, Patient Edition. New York, NY: SCID-I/P (2002).

[32] Fukunaga R Brown JW Bogg T. Decision making in the Balloon Analogue Risk Task (BART): anterior cingulate cortex signals loss aversion but not the infrequency of risky choices. Cognit Affect Behav Neurosci. (2012) 12:479–90. 10.3758/s13415-012-0102-1 22707378 PMC3493559

[33] Ashburner J Friston KJ. Unified segmentation. Neuroimage. (2005) 26:839–51. 10.1016/j.neuroimage.2005.02.018 15955494

[34] Liu X Hairston J Schrier M Fan J. Common and distinct networks underlying reward valence and processing stages: a meta-analysis of functional neuroimaging studies. Neurosci Biobehav Rev. (2011) 35:1219–36. 10.1016/j.neubiorev.2010.12.012 21185861 PMC3395003

[35] Whiteside SP Lynam DR The five factor model and impulsivity: Using a structural model of personality to understand impulsivity. Personal Individ Differ. (2001) 30:669–89. 10.1016/S0191-8869(00)00064-7

[36] Wilbertz T Deserno L Horstmann A Neumann J Villringer A Heinze HJ Response inhibition and its relation to multidimensional impulsivity. Neuroimage. (2014) 103:241–8. 10.1016/j.neuroimage.2014.09.021 25241087

[37] Domenech P Koechlin E Executive control and decision-making in the prefrontal cortex. Curr Opin Behav Sci. (2015) 1:101–6. 10.1016/j.cobeha.2014.10.007

[38] MacDonald S Mann R Chipman M Pakula B Erickson P Hathaway A Driving behavior under the influence of cannabis or cocaine. Traffic Injury Preven. (2008) 9:190–4. 10.1080/15389580802040295 18570139

[39] Smiley A Marijuana: on-road and driving-simulator studies. In: Kalant H Corrigall W Hall W Smart R, editors. The Health Effects of Cannabis Addiction Research Foundation. Toronto, ON: Centre for Addiction and Mental Health (1999). p. 173–91. Available online at: https://komornlaw.com/wp-content/uploads/2018/03/the-health-effects-of-cannabis-9780888683250.pdf

[40] Kalivas PW Volkow ND. The neural basis of addiction: a pathology of motivation and choice. Am J Psychiatry. (2005) 162:1403–13. 10.1176/appi.ajp.162.8.1403 16055761

[41] Sunsay C Rebec GV. Real-time dopamine efflux in the nucleus accumbens core during Pavlovian conditioning. Behav Neurosci. (2008) 122:358. 10.1037/0735-7044.122.2.358 18410174 PMC2664557

[42] Day JJ Jones JL Wightman RM Carelli RM. Phasic nucleus accumbens dopamine release encodes effort-and delay-related costs. Biol Psychiatr. (2010) 68:306–9. 10.1016/j.biopsych.2010.03.026 20452572 PMC2907444

[43] Sugam JA Day JJ Wightman RM Carelli RM. Phasic nucleus accumbens dopamine encodes risk-based decision-making behavior. Biol Psychiatr. (2012) 71:199–205. 10.1016/j.biopsych.2011.09.029 22055017 PMC3253943

[44] Saddoris MP Cacciapaglia F Wightman RM Carelli RM. Differential dopamine release dynamics in the nucleus accumbens core and shell reveal complementary signals for error prediction and incentive motivation. J Neurosci. (2015) 35:11572–82. 10.1523/JNEUROSCI.2344-15.2015 26290234 PMC4540796

[45] Scott JC Slomiak ST Jones JD Rosen AF Moore TM Gur RC. Association of cannabis with cognitive functioning in adolescents and young adults: a systematic review and meta-analysis. JAMA Psychiatr. (2018) 75:585–95. 10.1001/jamapsychiatry.2018.0335 29710074 PMC6137521

