# Supplementary material for: Does Chronic Cannabis Use Impact Risky Decision-Making: An Examination of fMRI Activation and Effective Connectivity?
Source: Front Psychiatry. 2020 Nov 27;11:599256. doi: 10.3389/fpsyt.2020.599256 (PMC7728610; doi:10.3389/fpsyt.2020.599256)

**Supplementary Data**

Figure S1: CB users showing differential activation in the right lateral posterior prefrontal cortex compared to non-users – p<0.01, k>100.


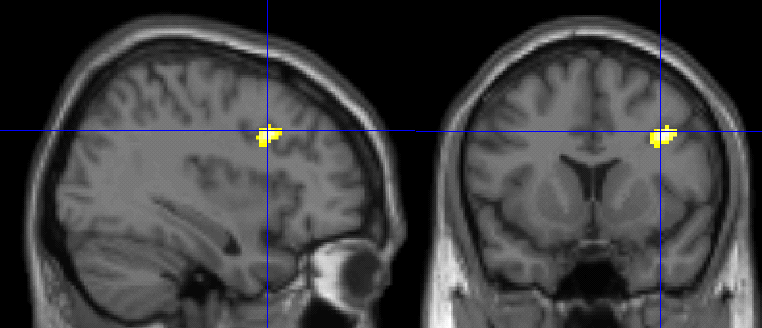

Supplement: Supplementary file 1 [file Data_Sheet_1.docx]
